# Methicillin-Resistant Staphylococcus aureus Eradication and Decolonization in Children Study (Part 2): Patient- and Parent-Centered Outcomes of Decolonization

**DOI:** 10.2196/14973

**Published:** 2020-05-20

**Authors:** Courtney M Moore, Sarah E Wiehe, Dustin O Lynch, Gina EM Claxton, Matthew P Landman, Aaron E Carroll, Paul I Musey

**Affiliations:** 1 Research Jam The Indiana Clinical and Translational Sciences Institute Indianapolis, IN United States; 2 Children's Health Services Research Department of Pediatrics Indiana University School of Medicine Indianapolis, IN United States; 3 Department of Surgery Indiana University School of Medicine Indianapolis, IN United States; 4 Pediatric and Adolescent Comparative Effectiveness Research Department of Pediatrics Indiana University School of Medicine Indianapolis, IN United States; 5 Department of Emergency Medicine Indiana University School of Medicine Indianapolis, IN United States

**Keywords:** patient-centered outcomes, Staphylococcus aureus, abscess, decolonization, human-centered design

## Abstract

**Background:**

Skin and soft tissue infections (SSTIs) due to community-acquired methicillin-resistant *Staphylococcus aureus* (MRSA) can lead to a number of significant known medical outcomes including hospitalization, surgical procedures such as incision and drainage (I&D), and the need for decolonization procedures to remove the bacteria from the skin and nose and prevent recurrent infection. Little research has been done to understand patient and caregiver-centered outcomes associated with the successful treatment of MRSA infection.

**Objective:**

This study aimed to uncover MRSA decolonization outcomes that are important to patients and their parents in order to create a set of prototype measures for use in the MRSA Eradication and Decolonization in Children (MEDiC) study.

**Methods:**

A 4-hour, human-centered design (HCD) workshop was held with 5 adolescents (aged 10-18 years) who had experienced an I&D procedure and 11 parents of children who had experienced an I&D procedure. The workshop explored the patient and family experience with skin infection to uncover patient-centered outcomes of MRSA treatment. The research team analyzed the audio and artifacts created during the workshop and coded for thematic similarity. The final themes represent patient-centered outcome domains to be measured in the MEDiC comparative effectiveness trial.

**Results:**

The workshop identified 9 outcomes of importance to patients and their parents: fewer MRSA outbreaks, improved emotional health, improved self-perception, decreased social stigma, increased amount of free time, increased control over free time, fewer days of school or work missed, decreased physical pain and discomfort, and decreased financial burden.

**Conclusions:**

This study represents an innovative HCD approach to engaging patients and families with lived experience with MRSA SSTIs in the study design and trial development to determine meaningful patient-centered outcomes. We were able to identify 9 major recurrent themes. These themes were used to develop the primary and secondary outcome measures for MEDiC, a prospectively enrolling comparative effectiveness trial.

**Trial Registration:**

ClinicalTrials.gov NCT02127658; https://clinicaltrials.gov/ct2/show/NCT02127658

## Introduction

### Background

Community-acquired skin and soft tissue infections (SSTIs) such as cellulitis, boils, myositis, and abscesses caused by antibiotic-resistant bacteria known as methicillin-resistant *Staphylococcus aureus* (MRSA) have risen dramatically over the past 20 years, and a significant proportion of these affect otherwise healthy children [1-8]. The estimated incidence of hospitalizations due to MRSA SSTIs is more than 45 per 100,000 children, with many children requiring surgical procedures such as incision and drainage (I&D), to drain pus caused by the infection [6,9,10]. Even with appropriate treatment, the rate of recurrent infection can be as high as 72% [11-16]. Thus, strategies are needed to reduce the rate of recurrent infection and comorbid suffering, cost, and health care utilization. One strategy, called *decolonization*, focuses on eradicating the presence of bacteria on the skin and in the nose of people at risk of infection. Examples of accepted decolonization protocols include the use of topical mupirocin (antibiotic) ointment in the nose to eliminate nasal carriage and chlorhexidine or bleach baths to eliminate skin carriage [17-19].

### Objectives

The MRSA Eradication and Decolonization in Children (MEDiC) comparative effectiveness trial [20] aimed to assess the effectiveness of 2 interventions: (1) abscess surgery and hygiene education compared with (2) abscess surgery and hygiene education followed by decolonization. Along with clinical measures, the study team aimed to understand the effects of these interventions on patient-centered outcomes. However, during the planning phase of our study, literature on patient-centered outcome measures for MRSA infection treatment was very limited. To uncover patient-centered outcomes that might be measured, we engaged patients with lived experiences with MRSA SSTIs and their caregivers in a human-centered design (HCD) workshop. This paper will discuss the workshop activities and results and how we incorporated these patient-centered outcomes into our overall study. The authors do not see this work as a final product, but rather a first step in inspiring the creation of a comprehensive set of MRSA patient-centered outcomes that can be measured alongside clinical outcomes in future work. A companion paper discusses a separate objective of this project, which was to engage patients and their families in the design of an MRSA decolonization toolkit to support families in the MEDiC study in adhering to the decolonization process [21].

## Methods

### Overview

The Indiana University School of Medicine Institutional Review Board approved this study. Participants were invited to participate in the workshop if they were a parent of a patient (3 months-18 years) who had undergone an I&D procedure at Riley Hospital for Children or were a patient (9-18 years) who had undergone an I&D procedure. All study participants (advisors) provided written informed consent, and children aged 9 to 13 years provided assent. All participants received US $20 per hour for their time and participation. The team hosted a 4-hour workshop that was audio-recorded.

### Human-Centered Design

HCD utilizes participatory methods of engagement to empower people to share their experiences, express their thoughts, and generate new ideas through what they say, do, and make [22]. *Say* methods include guided discussion, interviews, or questionnaires and elicit explicit information. *Do* methods include observation and elicit information that can be directly viewed. *Make* methods include collage, drawing, and 3D modeling and—through the maker’s explanation of their creation—elicit tacit information (or information that is known but difficult to access and express). When used in combination, these participatory methods help to ensure valuable involvement and can uncover the unmet needs of stakeholders [22]. Research Jam (RJ), the patient engagement core of the Indiana Clinical and Translational Sciences Institute, applies an HCD approach in the context of health research, collaborating with principal investigators such as the MEDiC study’s PM. HCD was chosen because its participatory methods— *make* methods in particular—are useful in helping participants create symbolic expressions of their tacit knowledge about the topic and provide a scaffolding from which participants can speak about their experiences. Patient and parent-centered outcomes, particularly those that are not immediately observable, but are *below the surface* are by their nature tacit. The study team was particularly careful to include activities that made space for the adolescents, who might feel uncomfortable speaking up in the more discussion-based portions of a workshop filled with adults. Activities that were first completed independently and then shared with the rest of the group allowed all participants to respond and share equally. This is important because research shows that parent reporting of adolescent outcomes may differ from self-reported adolescent outcomes, meaning that parent reporting ought not to be substituted for self-reporting [23]. In addition, we wanted to understand the personal experiences of the parent advisors, as we assumed MRSA affects them differently than it does their children. In this spirit, we aimed to gather self-reported experiences from adolescent advisors as well as the unique self-reported experiences of parent advisors themselves (as opposed to their interpretation of their children’s experiences). Participatory HCD methods were used to help both patient and parent advisors express unearthed outcomes of MRSA decolonization in an effort to, ultimately, create a patient-centered outcomes measurement tool.

### The Workshop

The research team held a 4-hour workshop (with a 30-min break for lunch) to explore a series of topics. One of these was the patient- and parent-centered outcomes of MRSA treatment. A total of 5 patient advisors who had undergone an I&D procedure attended the workshop. There were 3 females aged 10, 14, and 18 years and 2 males aged 14 and 18 years. In total, 11 parent advisors attended the workshop. This included 4 female parents of the adolescents (1 parent brought 2 children) and 7 additional parents (6 females and 1 male) of younger children (aged 15 months to 5 years) who had also experienced I&D. The patient advisors remained with their parents throughout the workshop. The 2 activities, *Fill-in-the-blank* and *Collage*, were utilized to gain an understanding of the outcomes of importance to patients and parents.

#### Fill-in-the-Blank

**Figure 1:**
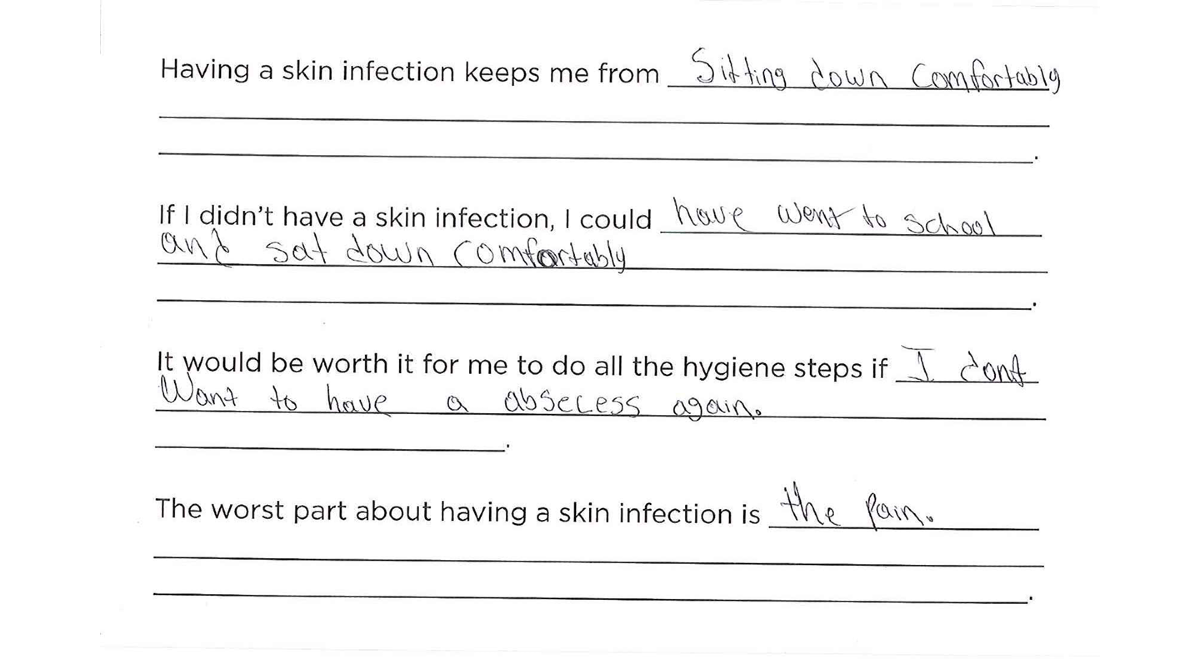
Patient advisor fill-in-the-blank discussing discomfort from methicillin-resistant Staphylococcus aureus.

Fill-in-the-blank is a *say* method that utilizes writing. Advisors were given a worksheet (Figure 1) with 4 fill-in-the-blank statements. The worksheets included the following statements:

1. Having a skin infection keeps me from ______________.
2. If I didn’t have a skin infection, I could ______________.
3. It would be worth it for me to do all the hygiene steps if _____________.
4. The worst part about having a skin infection is ____________.

Parent worksheets included the following statements:

1. Having a skin infection keeps my child from ___________.
2. My child having a skin infection keeps me from _____________.
3. If my child didn’t have a skin infection, we could _____________.
4. It would be worth it for me to do all the hygiene steps if ___________.

Facilitators asked patient and parent advisors to share their answers and prompted follow-up questions to encourage discussion within the group.

#### Collage

**Figure 2:**
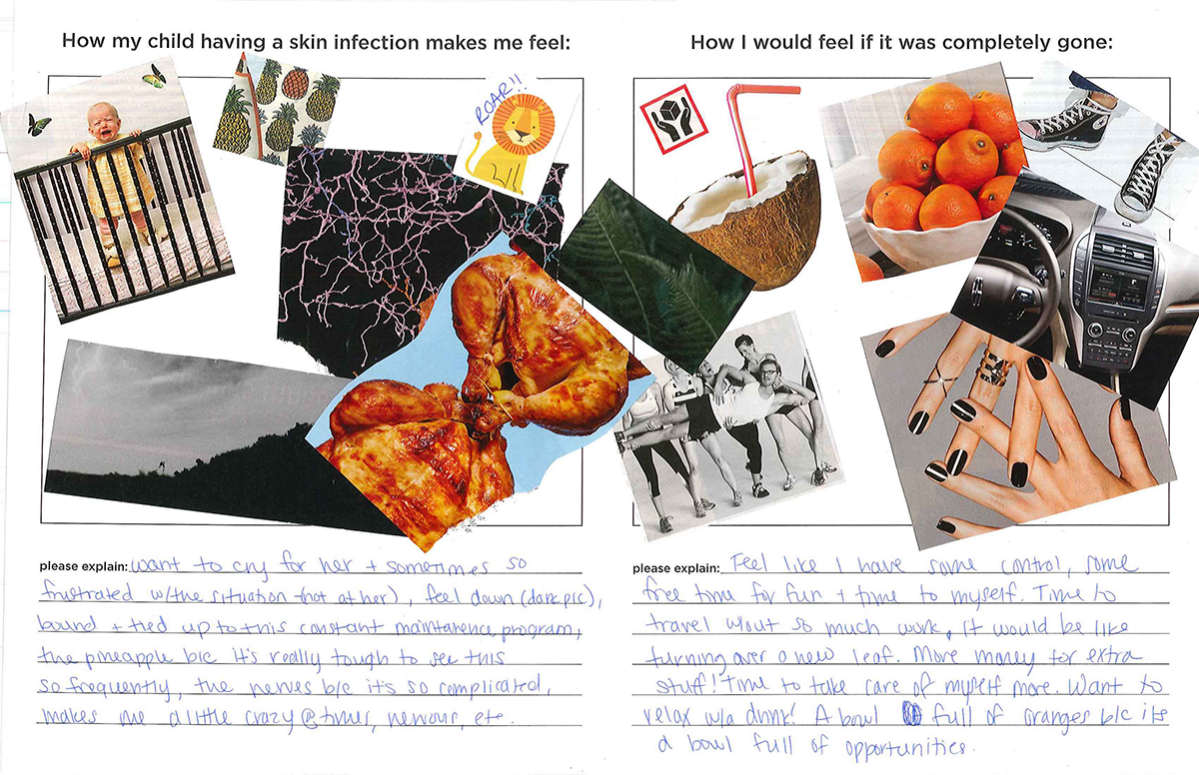
Parent advisor collage.

Collage is a *make* method in which the participant is asked to express their thoughts and feelings using images rather than strictly words. This approach allows participants to express themselves where words may fail, it aids in common understanding through the utilization of symbols and metaphors and reveals relationships that are harder to uncover through verbal or written presentations [24]. Participants are first given a large and varied selection of images not directly related to the topic of discussion and asked to create a collage that responds to a given prompt. They are then asked to write a brief explanation of the images they chose and how they relate to the topic of interest. Finally, they are asked to show their collage to the group and explain aloud why they chose the images they chose. The group is asked to respond to each participant by talking about parts of each collage they can relate to. During the workshop, a large selection of images, including both abstract and representative images, was placed in a pile in the center of the table. Advisors were given a large worksheet including 2 different prompts with empty space below. Each prompt asked the advisor to create a collage by gluing images of their choosing in the spaces provided. Below each collage space, blank lines were provided for the advisor to explain their collages (Figure 2). Patient advisors were given the prompts: *How having a skin infection makes me feel* and *How I would feel if it was completely gone*. Parent advisors were given the prompts: *How my child having a skin infection makes me feel* and *How I would feel if it was completely gone*. Facilitators asked advisors to share their worksheets with the group and prompted follow-up questions to encourage discussion within the group.

### Analysis

The analysis process for this research was inductive in nature and, specifically, based on Kolko’s methods for using the data, information, knowledge, wisdom framework, which describes the steps by which data (discrete symbols) are processed in stages to, ultimately, reach wisdom (development of increased value) [25]. As the ultimate goal of this research was to describe patient-centered outcome domains, the level of processing of our data stops at the knowledge stage. Responses from the fill-in-the-blank and collage worksheets were transcribed, and discrete ideas were separated into individual snippets of information. Additionally, discrete ideas from the audio-recorded discussion that added additional details were transcribed and added as individual snippets. In total, 2 members of the research team (CM and DL) who attended the workshop utilized affinity clustering to collaboratively organize the snippets. This method is an iterative process for grouping data by relationship (typically similarity) to move from data to information [25]. Once revisions to the clusters were finished, each cluster was then reviewed for content, discussed, and given a name to represent the theme of the snippets it contained. From here, a descriptive phrase was created for each theme to communicate its meaning to others.

## Results

### Workshop Participation

In total, 16 advisors attended the workshop, and 5 of these were adolescents (3 females aged 10, 14, and 18 years; 2 males aged 14 and 17 years). In total, 11 parents attended (10 females and 1 male; 4 parents of the adolescents and 7 parents of children aged 15 months to 5 years). Adolescents and their parents were kept together during the workshop.

### Key Themes

The following are the key themes related to outcomes of importance to patients with MRSA and their parents. Some of the key themes were mentioned by patient advisors, some by parent advisors, and many by both. This is indicated in brackets next to the theme in the descriptions below. This does not indicate importance. For example, although parent advisors did not report pain or discomfort as often as patient advisors, this does not indicate that their child’s discomfort is unimportant. Parent advisors were often explicitly asked to report about their own perspectives, emotions, and needs. The fill-in-the-blank worksheet, for example, asked about how their child’s skin infection limited their child as well as how it limited them. The collage worksheet asked about their own feelings rather than their child’s feelings. Table 1 shows an example of the process by which the team arrived at these themes.

**Table 1:** Process to move from quotes to cluster to theme.

Exemplary quotes	Cluster	Theme
“Having a skin infection keeps me from showing off too much of my skin.” “If my skin infection was gone, I’d feel beautiful…” “Having a skin infection makes me feel old.”	MRSA^a^ keeps me from feeling attractive and healthy	Improved self-perception
“Having a skin infection keeps my child from being their own self.” “If I didn’t have a skin infection, I could wear what I want.”	MRSA keeps the patient from being his or her self	Improved self-perception
“Older kids may not want to join sports things because of the worry. It would give them the freedom to say, ‘Hey, I want to go out for this sports team and I’m not going to be scared that I’ll have an outbreak and I’m not going to be able to play or people will think I’m weird because of this infection.’” “Having a skin infection keeps my child from feeling like the rest [of the girls at ballet] – having to wear [long sleeved leotards to cover her infection when the rest of the girls wear short sleeves].”	MRSA makes my child feel out of place	Improved self-perception

^a^MRSA: methicillin-resistant *Staphylococcus aureus.*

#### Fewer Methicillin-Resistant Staphylococcus aureus Outbreaks (Patient and Parent Advisors)

Patient and parent advisors ultimately wanted to experience fewer skin infection outbreaks. This was a very common answer for the question: *It would be worth it for me to do all the hygiene steps if _________.* Some advisors reported that they would do all of the hygiene steps for any degree of improvement, whereas others thought it would be worth it if they had no more outbreaks at all (Figure 1).

#### Decreased Physical Pain and Discomfort (Patient Advisors)

The patient advisors wrote and talked about the discomfort they felt from their skin infections. One advisor wrote: *“*Having a skin infection keeps me from sitting down comfortably” (Figure 1)*.* Other patient advisors mentioned episodes of intense itching. Parent advisors whose children were too young to put their discomfort into words were very interested in hearing from the patient advisors about what their skin infections felt like as this gave them some idea of how much discomfort their own children might be experiencing.

#### Improved Emotional Health (Patient and Parent Advisors)

Outbreaks from skin infections cause negative emotions such as stress, anger, and sadness. One patient advisor wrote about his collage: “[Having a skin infection] makes me feel like I wanna blow up."

**Figure 3:**
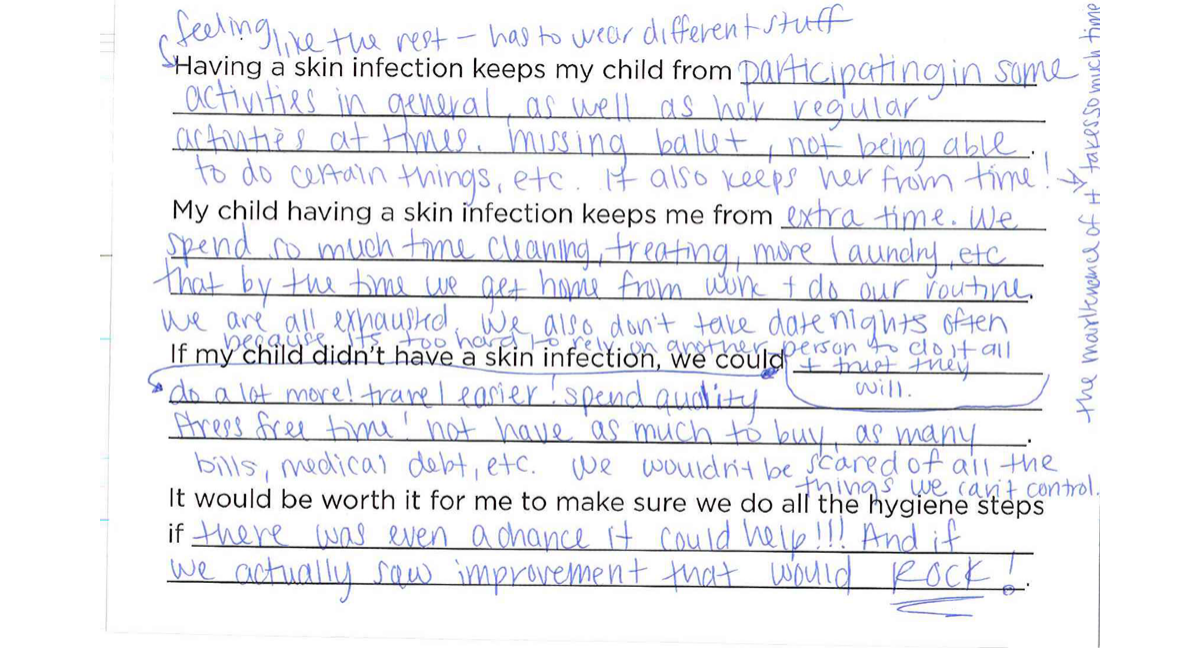
Parent advisor fill-in-the-blank.

One parent wrote about her collage: “[I chose the image of nerves] because it’s so complicated, makes me a little crazy at times, nervous, etc.” Getting rid of the infection would result in “Simple peace of mind…because if we see a spot, we’re just stressed.” Parent advisors reported that they were constantly alert for new spots that could potentially lead to another long course of treatment: “One little pimple is not one little pimple. It could turn into a full-blown softball and then we’re down at the hospital.” In many of the collages, advisors wrote that if their skin infections were completely gone, they would feel calm, relaxed, or happy. As one parent wrote in the fill-in-the-blank activity: “[If my child didn’t have a skin infection, we] wouldn’t be scared of all the things we can’t control.” (Figure 3)*.*

#### Improved Self-Perception (Patient and Parent Advisors)

Patient advisors mentioned that having a skin infection made them feel “dirty” or “not clean.” One patient advisor wrote in her fill-in-the-blank activity about how her skin infection affected her ability to express herself through her appearance:

[Having a skin infection] keeps me from showing off too much of my skin. [If I didn’t have a skin infection, I could] wear what I want and do what I want.

In her collage activity, she wrote about her skin infection’s effect on how she sees herself:

When having a skin infection, I feel…old. When the skin infection’s completely gone, I feel beautiful.

One mother talked about how her daughter stood out at ballet because she wore a long sleeve leotard to cover her skin infection whereas the other girls all wore short sleeves.

#### Decreased Social Stigma (Patient and Parent Advisors)

Patient and parent advisors described stigma associated with skin infections such as MRSA. As 1 parent advisor explained:

Older kids may not want to join sports things because of the worry. It would give them the freedom to say, “Hey, I want to go out for this sports team and I’m not going to be scared that I’ll have an outbreak and…people will think I’m weird because of this infection.”

Stigma did not only affect the patients but also their parents. One parent advisor shared a story about discovering, just days after hosting children for her daughter’s birthday party, that her daughter had MRSA. When she contacted the parents of the other children who attended to let them know, a few were angry and asked her why she had planned a party when her daughter had MRSA. She felt that these other parents were accusing her of being negligent, but she insisted that she would never have exposed anyone’s children to MRSA had she realized that her daughter was infected.

#### Increased Amount of Free Time (Parent Advisors)

Managing a skin infection such as MRSA takes up a lot of time. Parent advisors especially commented on the lack of free time they experienced due to all of the cleaning, sanitizing, laundry, visits to the doctor, trips to the emergency department, etc. One parent advisor wrote about her collage:

[If the infection were gone, I would feel] like I have some free time for fun and time to myself…[I chose the image of ] a bowl full of oranges because [my child not having a skin infection would be] a bowl full of opportunities.

#### Increased Control Over Free Time (Patient and Parent Advisors)

Patient and parent advisors reported that MRSA keeps them from participating in activities they enjoy. As 1 parent advisor explained in the fill-in-the-blank activity: “[Having a skin infection keeps my child from] participating in her regular activities at times [like] missing ballet.” In particular, parent advisors frequently mentioned wanting to be able to take their children to public pools but feeling that they could not because of the risk of their child infecting others. There was a lively discussion about whether or not chlorine in pools would keep the infection from spreading to others:

I’ve got a pool and we’re going to put it up, but it’ll have chlorine in it…I mean chlorine is bleach right? Does that help? Because that would determine whether or not we let other kids come over to swim in our pool.

Discomfort during an outbreak also kept some of the children from going to school or playing with friends and siblings. In addition, 1 married couple from the parent advisor group reported that the difficulty of explaining MRSA protocols to babysitters had kept them from going on their usual date nights as a couple: “We don’t go on date nights often because it’s too hard to rely on another person to do [the whole routine].” Traveling, in general, was something many families avoided while they were implementing decolonization protocols. To complete bleach baths during a vacation, for example, families would need to ensure there would be a bathtub available where they were staying and would need to bring bleach and measuring equipment (or purchase these upon arrival). The complications of travel during decolonization led many families to avoid it entirely.

#### Fewer Days of School or Work Missed (Patient and Parent Advisors)

Advisors reported that they sometimes had to miss school and work due to skin infection outbreaks. The patient advisors discussed having to miss school because their skin infection outbreaks made them too uncomfortable to concentrate. Parent advisors discussed missing work and their child missing school to receive medical care for outbreaks.

#### Decreased Financial Burden (Parent Advisors)

Parent advisors reported that caring for MRSA infections takes a lot of financial resources. There are medical bills from visits to the doctor, I&D procedures, emergency department visits, and prescriptions. In addition, there are added costs for purchasing bleach and other supplies and increased bills for water and electricity from extra baths and laundry. These additional costs are compounded by lost wages from parents missing work to take their children to receive care during work hours. These costs add up quickly and create additional burdens on families. One parent advisor wrote in her fill-in-the-blank activity: “If my child didn’t have a skin infection, we could not have as much to buy, as many bills, medical debt, etc.” (Figure 3).

## Discussion

### Development of Patient-Centered Outcome Measures

Outcomes research is increasingly incorporating the patient’s perspective in the study design and development of outcome measures [26-29]. This is thought to improve the credibility of research results and can be considered an ethical imperative [30]. Although the effectiveness of the 2 MRSA interventions in preventing recurrence was important, it was also important to determine what outcomes patients and families most desired. Thus, the initial step in our study design was to engage patients and families to uncover outcomes of importance when it comes to MRSA decolonization. This study represents what appears to be the first attempt to engage patients with MRSA SSTIs in study design and tailor the measures to fit with patient-centered outcomes. Through patient and family engagement in this study, we were able to identify 9 major themes along which we could craft methods of assessment as shown in Table 2.

**Table 2:** Patient-centered outcome themes and associated measures.

Key patient-centered outcome themes	Trial assessment measure
Fewer MRSA^a^ outbreaks	SSTIs^b^ recurrence by parental report at 6 weeks, 6 months, and 12 months Repeat surgical (incision and drainage) procedure by parental report at 6 weeks, 6 months and 12 months
Decreased physical pain and discomfort	Participant’s level of pain and discomfort at incision site via NRS^c^ pain scale at enrollment and 6 weeks
Improved emotional health, improved self-perception, decreased social stigma, increased amount of free time, and increased control over free time	Participants’ quality of life measured by parent-proxy report (or youth tool) of the PedsQL^d^ 4.0 at recruitment, 6 weeks, 6 months, and 12 months Estimated weekly time to adhere to intervention by parental report at 6 weeks
Fewer days of school or work missed and decreased financial burden	Participants’ school attendance by parental report at 6 weeks, 6 months, and 12 months Assessment of parents’ work attendance by self-report at 6 weeks, 6 months, and 12 months

^a^MRSA: methicillin-resistant *Staphylococcus aureus*.

^b^SSTIs: skin and soft tissue infections.

^c^NRS: numeric rating scale.

^d^PedsQL: Pediatric Quality of Life Inventory.

A number of these themes are interrelated, and thus the methods of assessment overlap.

The number of recurrent MRSA outbreaks was the most common theme of importance to patient advisors and parent advisors. This also happened to be the a priori primary clinically related outcome planned by the investigators. With rates of MRSA recurrence of over 70% [11-16] in some cases, it makes sense from a clinical- and patient-centered perspective that this outcome would be of paramount importance to assess in our study. Thus, we defined our primary outcome of interest as the proportion of participants with recurrent SSTIs by parental report. This would first be assessed at 6-weeks postintervention. This is a standard timeframe for assessment in emergency medicine practice and would also provide an early opportunity to check-in on study participant protocol compliance. This outcome was also assessed at 6 and 12 months. Additionally, after engaging with the participants, we believed that although avoiding infection recurrence was ideal, the avoidance of a repeat surgical procedure seemed to touch many of the key themes of importance. Thus, we also defined a secondary outcome as the proportion of participants requiring repeat I&D procedures by parental report at 6 weeks, 6 months, and 12 months. The desire for decreased pain and physical discomfort was also a major theme extracted from our interaction. Although these are partially assessed via the repeated infection/surgical intervention outcomes described earlier, we decided to assess the level of pain and discomfort at the original surgical incision site via a patient/parent report using the numeric rating scale [31] at enrollment and again at 6 weeks.

The quality of life of the patient appeared to be at the heart of the next 5 extracted themes: (1) improved emotional health, (2) improved self-perception, (3) decreased social stigma, (4) increased amount of free time, and (5) increased control over free time. Regarding improved self-perception, the literature on self-esteem identifies the self-concept of appearance as the most influential aspect affecting overall self-esteem [32]. In addition, pediatric skin diseases, particularly acquired, visible diseases such as acne or hidradenitis suppurativa, have a high negative impact on school-age and adolescent self-esteem, partly due to poor self-concept of appearance and social stigma. These diseases are associated with increased depression and suicidal ideation among older children and teens [33]. Although data on the impact of MRSA on these outcomes are lacking, we suspect that there are similarities to other skin diseases with similar features (eg, acquired and visible). How best to assess these outcomes among a pediatric population ranging in age from 3 months to 18 years of age was debated between the investigators. Ultimately, it was decided that these themes could not reliably be assessed via custom items given the age range of the participants and that an accepted and validated tool would be needed. Thus, the Pediatric Quality of Life Inventory was chosen as it is a health-related quality of life measurement tool for both healthy children and those with acute and chronic health issues [34]. This validated multidimensional tool features both self-report (5 to 18 years of age) and parent proxy-report (2 to 18 years of age) and assesses physical, emotional, social, and school functioning through 3 scores: a total score, a physical health summary score, and a psychosocial health summary score. Additionally, we felt that assessment of the time required to adhere to the study interventions would be an important measure of the burden on patients and their families.

The final themes extracted from our engagement were the desire for fewer days of school and work missed as well as a decreased financial burden. Similar to quality of life, these have not been assessed in the context of outpatient decolonization protocols. The literature on the economic costs of MRSA and other skin infections outside of direct health care system costs is limited. However, a study of the direct and indirect costs of surgical site infections (including MRSA) in adults in Spain found that 90% of surgical site infection costs were indirect costs outside of the health care system, such as lost productivity from missed work days or time spent by a family member attending to the patient [35]. Days of school and work missed secondary to dealing with SSTIs are straightforward and easy to collect via self-report. Other elements of financial burden, however, are more difficult to assess directly given the expected variability in cost/charges for medical care, insurance status, baseline socioeconomic status, and more granular issues such as the lack of a home washer and dryer requiring laundromat visits. However, it was felt that the days of school/work missed was an appropriate surrogate marker for financial burden that was feasible for the study to assess. In many cases, keeping a child home from school for illness or the circumstances surrounding a hospitalization requires at least one parent to take time away from their job to attend to the child. This could mean lost wages or arranging for childcare and potentially incurring unexpected costs.

MRSA patient-centered outcomes, to our knowledge, have yet to be explored. The literature related to quality of life or patient-centered outcomes for other skin conditions show that skin conditions do have an effect on quality of life in ways similar to our findings. For example, in 1 study, adults with atopic dermatitis reported that their condition causes avoidance of social interactions and impacts their activities [36]. A literature review related to the psychosocial effects of various chronic skin conditions found that psoriasis, a condition that causes red, scaly, painful patches of skin, impacts work, relationships, and social activities and causes anxiety and depression not only for the patient but also for cohabitants [37]. MRSA infection causes symptoms that are similar to those caused by other skin conditions, such as painful and visible skin lesions, but it also has unique aspects, particularly related to the burden of treatments such as decolonization. Thus, MRSA-specific outcomes should be measured.

### Follow-Up

In total, 5 parent advisors participated in a small follow-up survey that asked them to check which of the patient-centered outcomes as described earlier were important to them when it came to MRSA treatment. In all, 3 of the 5 advisors indicated that all of the patient-centered outcomes were important to them. Items that did not receive unanimous endorsement were *improved emotional health*, *improved self-perception*, *decreased social stigma*, *increased amount of free time*, and *decreased financial burden*. This small follow-up suggests that the patient-centered outcomes uncovered during this study are promising, but additional work will be required to validate them in a larger population.

### Limitations

Although these outcomes were codeveloped with a small sample size of patients and parents, all of the patients and parents had experience with skin infections such as MRSA, and a few had previously utilized or were currently utilizing bleach bath protocols. In addition, parents with children ranging from 15 months to 18 years of age and adolescents ranging from 10 to 18 years of age attended the workshop. This allowed for a range of perspectives based on patient age. HCD participatory methods also help to mitigate small numbers by getting to deeper information more quickly and allowing for discussion from the group that builds on the ideas brought up by individual members.

HCD has the same limitations as other qualitative research approaches, such as findings not being statistically representative or generalizable and potential for researcher bias in data collection and analysis. The first limitation is inherent in qualitative research and is why we see our patient-centered outcomes as a starting point that must be validated with future quantitative studies. The second was mitigated as much as possible by carefully considering the questions to be asked during our workshop and by completing analysis as a team, encouraging discussion and debate as pieces of data were grouped and theme descriptions determined.

One important limitation is that, due to limited staff resources, adolescents stayed in the room with their parents for the duration of the workshop. If adolescents had been separated from the adults, they might have been more vocal and brought up additional issues they may not have been comfortable discussing in front of their parents or other adults.

In addition, due to challenges in recruiting and retaining participants for the MEDiC trial, we were unable to effectively validate our findings or our measures or to fully assess these outcomes in our study population as planned.

### Conclusions

This study represents the first attempt to engage patients with MRSA SSTIs in study design and trial development using HCD to engage patients and their families with lived experiences to determine meaningful patient-centered outcomes. Through this crucial participation, we were able to identify 9 major recurrent themes. These themes were used to develop the primary and secondary outcome measures for MEDiC, a prospectively enrolling comparative effectiveness trial launched in February 2016. The authors do not see this work as a final product, but rather a first step in inspiring the creation of a comprehensive set of MRSA patient-centered outcomes that can be measured alongside clinical outcomes in future work.

## Abbreviations

HCD: human-centered design
I&D: incision and drainage
MEDiC: MRSA Eradication and Decolonization in Children
MRSA: methicillin-resistant Staphylococcus aureus
SSTI: skin and soft tissue infection
